# Transcriptome signatures from discordant sibling pairs reveal changes in peripheral blood immune cell composition in Autism Spectrum Disorder

**DOI:** 10.1038/s41398-020-0778-x

**Published:** 2020-04-14

**Authors:** Michele Filosi, Tony Kam-Thong, Laurent Essioux, Pierandrea Muglia, Elisabetta Trabetti, Will Spooren, Bertram Müller-Myshok, Italian Autism Network, Giovanni Alibrio, Laura Anchisi, Milena Andruccioli, Arianna Benvenuto, Pier Antonio Battistella, Flavio Boscaini, Carmela Bravaccio, Elisa Ceppi, Diego Cosentino, Paolo Curatolo, Lucio Da Ros, Bernardo Dalla Bernardina, Andrea De Giacomo, Giuseppa Di Vita, Enrico Domenici, Massimo Elia, Filippo Gitti, Serenella Grittani, Anna Linda Lamanna, Elisa Mani, Barbara Manzi, Lucia Margari, Gabriele Masi, Massimo Molteni, Pierandrea Muglia, Franco Nardocci, Antonio Pascotto, Antonia Parmeggiani, Pier Franco Pignatti, Tiziana Piroddi, Paola Prandini, Emiliangelo Ratti, Paolo Rizzini, Sebastiano Russo, Renato Scifo, Raffaella Tancredi, Alessandra Tiberti, Elisabetta Trabetti, Leonardo Zoccante, Alessandro Zuddas, Enrico Domenici

**Affiliations:** 1 Department of Cellular, Computational and Integrative Biology (CIBIO), University of Trento, Trento (TN), Italy; 2 Roche Pharmaceutical Research and Early Development (pRED), Roche Innovation Center, Grenzacherstrasse 124, Basel, Switzerland; 3 UCB Pharma, Brussels, Belgium; 4 Department of Neurosciences, Biomedicine and Movement Sciences, Section of Biology and Genetics, University of Verona, Verona, Italy; 5 Max Planck Institute of Psychiatry, Munich, Germany; 6 Azienda Ospedaliera Spedali Civili di Brescia, Brescia, Italy; 7 Università degli Studi di Cagliari, Centro per lo Studio delle Terapie Farmacologiche in Neuropsichiatria dell’infanzia e dell’adolescenza, Cagliari, Italy; 8 Ospedale Infermi, Divisione Neuropsichiatria Infantile, Centro per l’autismo, Rimini, Italy; 9 Neuropsichiatria Infantile, Università Tor Vergata, Policlinico Tor Vergata, Roma, Italy; 10 Neurologia Pediatrica, Dipartimento di Pediatria, Università di Padova, Padova, Italy; 11 Servizio di Neuropsichiatria Infantile, Azienda Ospedaliera, Istituti Ospitalieri di Verona, Policlinico G.B. Rossi, Verona, Italy; 12 Neuropsichiatria Infantile - Seconda Università degli Studi di Napoli, Napoli, Italy; 13 IRCCS Eugenio Medea, Monza, Italy; 14 Fondazione SmithKline, Verona, Italy; 15 Neuropsichiatria Infantile, Università “Tor Vergata”, Policlinico Tor Vergata, Roma, Italy; 16 Fondazione FSK, Verona, Italy; 17 Servizio di Neuropsichiatria Infantile, Azienda Ospedaliera, Istituti Ospitalieri di Verona, Policlinico G.B. Rossi, Verona, Italy; 18 Unità Operativa di Neuropsichiatria Infantile, Dipartimento di Scienze Neurologiche e Psichiatriche, Università degli Studi Bari, Bari, Italy; 19 IRCCS Associazione Oasi Maria SS., Troina (Enna), Italy; 20 Department of Cellular, Computational and Integrative Biology (CIBIO), University of Trento, Trento, Italy; 21 IRCCS Associazione Oasi Maria SS., Troina (Enna), Italy; 22 Azienda Ospedaliera Spedali Civili di Brescia, Brescia, Italy; 23 Ospedale Infermi - Divisione Neuropsichiatria Infantile, Centro per l’autismo, Rimini, Italy; 24 Unità Operativa di Neuropsichiatria Infantile, Dipartimento di Scienze Neurologiche e, Psichiatriche, Università degli Studi di Bari, Bari, Italy; 25 IRCCS Eugenio Medea, Monza, Italy; 26 Neuropsichiatria Infantile, Università Tor Vergata, Policlinico Tor Vergata, Roma, Italy; 27 Unità Operativa di Neuropsichiatria Infantile, Dipartimento di Scienze Neurologiche e Psichiatriche, Università degli Studi di Bari, Bari, Italy; 28 IRCCS Stella Maris, Pisa, Italy; 29 IRCCS Eugenio Medea, Monza, Italy; 30 UCB Pharma, Brussels, Belgium; 31 Neuropsichiatria infantile, Modena, Italy; 32 Neuropsichiatria Infantile, Seconda Università degli Studi di Napoli, Napoli, Italy; 33 Dipartimento di Scienze Mediche e Chirurgiche, Università di Bologna, Bologna, Italy; 34 Universita’ di Verona, Verona, Italy; 35 Ospedale Infermi, Divisione Neuropsichiatria Infantile, Centro per l’autismo, Rimini, Italy; 36 Universita’ di Verona, Verona, Italy; 37 Central Nervous System Therapeutic Area Unit, Takeda, Boston, USA; 38 Fondazione FSK, Verona, Italy; 39 Neuropsichiatra Infantile, AUSL 3 Catania, Acireale (Catania), Catania, Italy; 40 Neuropsichiatra Infantile, AUSL 3 Catania, Acireale (Catania), Catania, Italy; 41 IRCCS Stella Maris, Pisa, Italy; 42 Azienda Ospedaliera Spedali Civili di Brescia, Brescia, Italy; 43 Department of Neurosciences, Biomedicine and Movement Sciences, Section of Biology and Genetics, University of Verona, Verona, Italy; 44 Servizio di Neuropsichiatria Infantile, Azienda Ospedaliera, Istituti Ospitalieri di Verona, Policlinico G.B. Rossi, Verona, Italy; 45 Università degli Studi di Cagliari, Centro per lo Studio delle Terapie Farmacologiche in Neuropsichiatria dell’infanzia e dell’adolescenza, Cagliari, Italy; 46 Fondazione The Microsoft Research - University of Trento Centre for Computational and Systems Biology (COSBI), Rovereto (TN), Italy

**Keywords:** Molecular neuroscience, Biomarkers

## Abstract

Notwithstanding several research efforts in the past years, robust and replicable molecular signatures for autism spectrum disorders from peripheral blood remain elusive. The available literature on blood transcriptome in ASD suggests that through accurate experimental design it is possible to extract important information on the disease pathophysiology at the peripheral level. Here we exploit the availability of a resource for molecular biomarkers in ASD, the Italian Autism Network (ITAN) collection, for the investigation of transcriptomic signatures in ASD based on a discordant sibling pair design. Whole blood samples from 75 discordant sibling pairs selected from the ITAN network where submitted to RNASeq analysis and data analyzed by complementary approaches. Overall, differences in gene expression between affected and unaffected siblings were small. In order to assess the contribution of differences in the relative proportion of blood cells between discordant siblings, we have applied two different cell deconvolution algorithms, showing that the observed molecular signatures mainly reflect changes in peripheral blood immune cell composition, in particular NK cells. The results obtained by the cell deconvolution approach are supported by the analysis performed by WGCNA. Our report describes the largest differential gene expression profiling in peripheral blood of ASD subjects and controls conducted by RNASeq. The observed signatures are consistent with the hypothesis of immune alterations in autism and an increased risk of developing autism in subjects exposed to prenatal infections or stress. Our study also points to a potential role of NMUR1, HMGB3, and PTPRN2 in ASD.

## Introduction

Autism Spectrum Disorder (ASD) is a group of neurodevelopmental disorders with onset in early childhood, characterized by a triad of core symptoms (impaired social interaction, poor language development and communication and repetitive and narrow pattern of behaviors and interests. The heritability of ASD is relatively well established, and it is now thought that 10–20% of the cases are genetically defined^1^. Yet, the genetic etiology is not fully understood, and genome-wide investigations support a complex genetic architecture based on major genes and polygenic factors having different extent of contribution across the ASD spectrum^2^. Several etiological hypotheses for ASD exist, as for example altered synaptic dysfunction leading to an imbalance of excitatory and inhibitory neurotransmission^3^, although a unifying etiological theory is still missing. Abnormalities in brain tissue at the molecular level, including transcriptional and splicing dysregulations, have been shown to correlate with neuronal dysfunctions^4–6^. The investigations on post-mortem tissue from ASD patients have shed light on the molecular mechanisms underlying the disorder at brain level, confirming the importance of transcriptional analysis in disease characterization. However, the search for a reliable molecular signature for ASD based on peripheral samples, which might help clinicians in early diagnosis and in the identification of ASD subgroups, is still ongoing. Several attempts in this direction have been performed by gene expression analysis of lymphoblastoid cell lines^7–15^ and blood samples^16–25^ (see for a review ref. ^26^). Overall, these studies suggest the implication of several signaling pathways and the immune response in ASD, but a consistent set of diagnostic biomarkers remains elusive. A recent meta-analysis of blood-based transcriptome investigations in ASD remarks the hypothesis of implication of the immunologic function^27^. Indeed, the predominant signature observed in the ASD blood transcriptome was characterized by reduced expression of transcripts related to innate immune and inflammatory signaling, including type I and type II interferon-stimulated signaling cascades. Additional findings were under-expression of EGF-, PDGF-, PI3K-AKT-mTOR-, and RAS-MAPK-signaling cascades, and over-expression of modules enriched in ribosomal translation and NK-cell-related functions.

The Italian Autism Network (ITAN) consists of a collection of families formed by ASD probands, unaffected siblings when available, and parents recruited through thirteen clinical centers across Italy^28^. The network has an associated repository of genomic DNA, blood RNA, plasma, and lymphoblastoid cell lines to enable integrated genetic and biomarker research.

Here we present the first transcriptome analysis conducted on the ITAN cohort aimed at identifying peripheral signatures for ASD. The study was conducted on a selected subset of the available discordant sibling pairs, consisting in a total of 150 subjects. Blood samples were subjected to RNA Sequencing and data were analyzed by exploiting a matched pairs design. In order to disentangle the complexity of the molecular changes potentially arising from differences in blood composition in disease vs healthy state, we applied two different cell deconvolution algorithms and validated our findings with a complimentary WGCNA analysis.

## Material and methods

### Participants

The ITAN collection comprehends more than 800 subjects belonging to 256 families recruited across thirteen centers^28^. The study protocol was in first instance approved by the Verona Hospital Ethical Review board (study protocol AUT-SFK001, CE1419), and subsequently by the Ethical Review Committees of each recruiting site. All adult subjects participating in this project gave their written consent (or the consent for their children); assent to participate to the study from the children was obtained whenever obtainable. Diagnosis of autism spectrum disorder according to DSM IV^29^, was assessed by experienced child psychiatrists using standard tools: Broader Phenotype Autism Symptom Scale (BPASS), Autism Diagnostic Interview—Revised (ADI-R), Autism Diagnostic Observation Schedule (ADOS), and Krug Asperger Disorder Index screening. We selected families with at least two siblings discordant for ASD diagnosis, with the affected children between 4 and 18 years of age. After the selection process, 75 sibling pairs were sequenced, for a total of 150 subjects. Demographic parameters, age, gender, and ethnicity for each subject are shown in Table 1.

**Table 1:** Demographic information on the subset of the ITAN collection used in this study.

Sibling pairs		Autism	PDD-NOS	ASP	Total
Gender concordance (N of subjects)	Male	27	5	6	38
	Female	4	1	0	5
	Discordant	24	4	4	32
Ethnicity (*N* of subjects)	CEU	41	8	6	55
	Other	14	2	4	20
Age	Cases	10.02	10.00	12.7	10.33
	CTRL	11.34	10.5	15.1	11.7

*PDD-NOS* Pervasive development disorder not otherwise specified, *ASP* Asperger syndrome.

### RNA sequencing

Total RNA from blood samples, randomized by Roche statisticians, was submitted to Poly-A RNA sequencing on Illumina RNASeq Platform. Pre-alignment/mapping quality control (using Illumina sequencing accuracy quality scoring to estimate the base calling error probabilities, thresholds of all bases >Q30) was performed to confirm that key laboratory quality metrics criteria were met. To estimate expression at gene level, paired-end RNAseq reads were mapped to the human genome (hg19) by using the short-read aligner GSNAP^30^. The number of mapped reads for all RefSeq transcript variants of a gene counts were combined into a single value by using SAMTOOLS^31^. Technical features such as RIN, sequencing batch number, sequencing lane and pool, RIN, µgRNA were collected for subsequent statistical analysis. The RNA Sequencing data that support the findings of this study are available from the ITAN Foundation (see https://www.fondazioneitan.org for details) upon submission of an official request. Data are released only for research purposes, upon assessment of a project proposal by the ITAN Scientific Committee.

### HLA typing and HLA and KIR variants expression from RNASeq

We inferred HLA genotypes and quantified HLA allele expression from RNA Sequencing data we used a recently developed computational pipeline called HLAPers^32^. The pipeline consists in two steps: (i) the HLA typing step, where reads are aligned on a curated database of HLA variants available as transcript sequences to infer the subject genotype; (ii) the quantification step, where expression is estimated based on the number of reads aligning to each reference, using a model accounting for the occurrence of reads mapping to multiple HLA references corresponding to different HLA alleles or genes. We thus realigned original ITAN RNASeq data onto the International Immunogenetics/HLA (IMGT) database release 3.31.0^33^ to extract highly specific HLA transcripts and, based on the best aligned sequences, inferred the subject genotype for the HLA variants available in the IMGT database.

For KIR genes, we used the gene counts combined into single value by using SAMTOOLS to quantify specific the expression of KIR genes, since the KIR sequences database^34^ does not provide transcript sequences for different genotypes.

### Statistical analysis

A diagram representing our full analysis workflow is shown in Supplementary Fig. 1. After the preprocessing step, we filtered genes with count-per-million (CPM) greater than 1 in 25% of the samples, then gene expression was normalized and log-transformed using functions implemented in edgeR^35^. To account for differences in sequencing depths and RNA composition across samples, gene counts were normalized using the trimmed mean normalization method, while for the estimation of the biological coefficient of variation (BCV) under the assumption of a negative binomial distribution we used the estimateDisp function from edgeR (see Supplementary Fig. 2 where the BCV estimate is reported). A preliminary multivariate analysis was conducted to identify data structure, outliers and other factors potentially affecting expression levels. We used the non-affected sibling as the control, thus exploiting the high degree of kinship with the ASD subject, by using a paired design to correct for sample dependence. We estimated the beta coefficients for each technical and demographic feature, including diagnosis, using a generalized linear model. The technical features included in the model are described in the previous section, while the demographic features are: gender, ethnicity, age, family id and diagnosis. A moderated gene-wise variance was then computed based on an empirical Bayes procedure^36^ to extract the differential gene expression (DGE) signature. The same model was also run by including an estimation score for blood cell composition for each sample (see below) as covariate in the DGE analysis. For all DGE analyses we used a paired design comparing the affected subjects with the related discordant siblings. All analyses were conducted with R 3.4.3.

Enrichment analysis of the differentially expressed genes (DEGs) was performed by using the online web-service KOBAS 3.0^37^. We run the analysis with default parameters on five human pathway databases (KEGG, BioCyc, Reactome, Panther, GO) using all blood expressed genes from our RNASeq dataset as background list for the enrichment. Additional enrichment analysis was conducted by using Open Target^38^ and StringDB^39^ to identify diseases, drugs and literature associated with the DEG list.

### Cell composition estimate

Two complementary deconvolution methods were used to estimate blood cell composition, ie CIBERSORT and xCell. CIBERSORT allows the extraction of cell mixture proportions from gene expression profiles based on support vector regression of a cell-specific signature matrix^40^. We have used the on-line version of the tool^41^, which allows the estimation of a cell mixture based on a signature matrix validated with curated signatures from 22 human hematopoietic cell types (LM22). The algorithm takes the gene expression profile matrix as an input and returns a proportion between 0 and 1 for the 22 cell types and an empirical *p*-value. xCell^42^ improves previous deconvolution algorithms by correcting the biases due to overlapping signatures between similar cell types. The signature matrix is based on lists of genes extracted from multiple gene expression analysis and validated by cell sorting studies. Differently from previous methods, xCell uses single sample enrichment to estimate a score linearly correlated with the abundances of cell types in the cell mixture. We adapted a R package publicly available on GitHub^43^ by using a subset of the original xCell signature consisting in 14 cell types relative to blood (based on the studies SDY311 and SDY420^44^ from the ImmPort database^45^). Input data of the cell enrichment algorithm was the voom normalized gene expression^46^. xCell returns a cell-specific enrichment score (linearly correlated with cell abundance in the mixture) and an associated FDR adjusted *p*-value. As suggested by the original paper^42^, we selected cell types with a corrected *p*-value < 0.2. For both methods, differences in cell composition between discordant siblings were assessed by *t*-test with matched pairs design.

### WGCNA

We followed the pipeline proposed by Langfelder et al.^44^ in their CRAN package to infer gene co-expression networks and identify network modules within R 3.4.3 statistical environment. Further details of the methods used throughout this analysis can be found in the original manuscript^47^ and on the website^48^. Networks were inferred using the TOMsimilarityFromExpr function with “bicor” as correlation measure. The soft-threshold parameter was optimized with the function pickSoftThreshold and the best threshold (α = 12) selected by visual inspection, as suggested by the WGCNA pipeline. Correlations between modules eigengenes, diagnosis and cell mixture estimation score were computed. Modules with the highest correlation and significant *p*-value (α < = 0.05) were selected for further analysis.

## Results

### Identification of DEGs

**Fig. 1:**
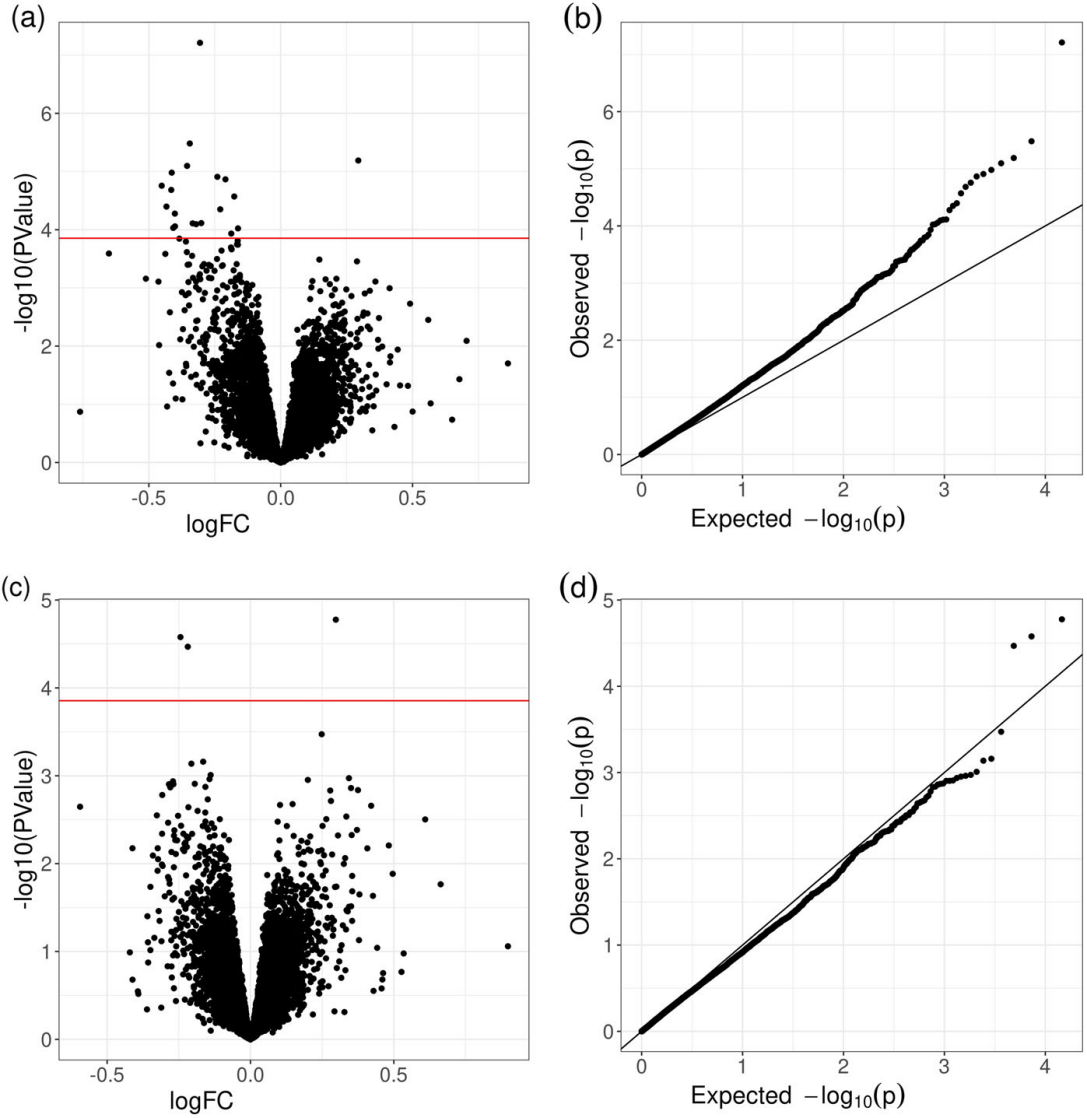
Differential gene expression analysis. Volcano plots and QQ-plots for the standard model (**a**, **b**) and for the model with cell composition score as covariate (**c**, **d**). The inclusion of cell enrichment scores in the DGE model results with a decrease of the inflation rate as measured by the lambda (lambda = 1.23 in (**b**); lambda = 0.975 in (**d**)).

An exploratory analysis of the RNASeq data through the PCA analysis did not reveal any noticeable outliers (see Supplementary Fig. 3). A close similarity of expression was observed for siblings, supporting the choice of a discordant sib-pair experimental design. When assessing the fraction of the variance explained by the first five PCA components for each covariate, the family component was indeed confirmed to be the predominant factor (see Supplementary Fig. 4 and Supplementary Table 1). The DGE analysis revealed subtle differences in expression between discordant siblings (see Supplementary Table 2 for the full list of DEGs). Setting a threshold on the FDR corrected *p*-value to 0.1 or 0.05 result with a shorter list of 21 and 10 genes, respectively (see Table 2a). The volcano plot in Fig. 1(a) shows that the DEGs are predominantly downregulated in ASD, in accordance with previous findings^18^. The QQ plot shows a deviation from the null model even at *p*-values below significance, with a calculated lambda value of 1.23, suggesting an inflation of the type I error rate, possibly due to hidden variability. Based on prior evidence of sex differences in blood transcriptomic signatures of ASD^15,49^, we performed a separate analysis for male sibling pairs, for which an adequate number was available (see Supplementary Fig. 5, and Supplementary Table 3). The results suggest a strong correlation between the gender-covariate analysis conducted on the full datasets and the male-specific analysis, with most genes (86%) maintaining the same direction of expression change, and no discordant sign for the first 900 genes of the ranked list of DGE in the full datasets (see also Supplementary Fig. 6).

**Table 2:** DGE model results ordered by FDR.

(a) Standard model				(b) xCell enrichment		
Name	LogFC	*P* Value	FDR	LogFC	*P* Value	FDR
HMGB3	−0.306	6.15e-08	**8.99e−04**	−0.250	2.02e-05	**1.54e−01**
NMUR1	−0.345	3.30e-06	**2.41e−02**	−0.224	2.11e-05	**1.54e−01**
PTPRN2	0.294	6.47e-06	**2.84e−02**	0.290	3.36e-05	**1.63e−01**
NKG7	−0.355	8.01e-06	**2.84e−02**	−0.207	6.71e-04	9.93e−01
PIF1	−0.413	105e-05	**2.84e−02**	−0.299	7.54e-04	9.93e−01
KLRD1	−0.240	1.23e-05	**2.84e−02**	−0.145	7.39e-04	9.93e−01
FKBP11	−0.210	1.36e-05	**2.84e−02**	−0.159	1.31e-03	9.93e−01
GLNY	−0.451	1.75e-05	**3.20e−02**	−0.302	5.76e-04	9.93e−01
CLIC3	−0.415	2.07e-05	**3.36e−02**	−0.269	1.69e-03	9.98e−01
MANF	−0.176	2.69e-05	**3.93e−02**	−0.141	1.36e-03	9.93e−01

(a) Results obtained by the paired design, including technical and demographic covariates (“standard model”).

(b) Results obtained by including in the standard model the cell composition score derived by cell deconvolution analysis with xCell as additional covariate. Only results for FDR < 0.05 from the standard model are shown in the table.

### Enrichment analysis

We performed enrichment analysis by submitting the full list of nominally significant DEGs to the KOBAS algorithm^37^. Enrichment analysis with KOBAS shows five statistically significant enriched pathways with FDR < 0.05 (See Supplementary Table 4). The first two pathways are Natural Killer-cell mediated cytotoxicity and immunoregulatory interactions between a Lymphoid and a non-Lymphoid cell, suggesting a dysregulation in the immune functions in ASD.

When submitting the list of significant genes at FDR < 0.25 to Open Target batch search to look for diseases associated to our signature, the top finding was cytomegalovirus infection (see Supplementary Table 5). Cytomegalovirus infection was still the top finding when restricting the analysis to the shorter 21 gene list (FDR < 0.1). Similarly, when searching for published signatures similar to our list of FDR < 0.25 significant genes (by using StringDB) we identified the enrichment in CMV-specific CD4+ T cells signatures from chronically infected healthy donors^50^ as the top hit (*P* value = 8.86 E−10).

### Cell composition

Despite the limited number of DEGs between discordant siblings, our enrichment analysis indicates a clear dysregulation in immune functions. To verify if such differences are related to differences in immune cell populations between the autism and control groups, we submitted the gene expression dataset to two complimentary methods to estimate cell composition in blood. By using CIBERSORT and the LM 22 signature matrix to estimate blood cell proportion, and comparing discordant siblings with a paired design, we were able to show statistically significant differences (*p*-value 0.05) for four cell types, including NK cells, Tgd, B cells and CD4+ TEM cells (see Table 3a). The results from the analysis with xCell are consistent with CIBERSORT, in particular for NK cells and B cells (see Table 3b). Indeed, we found high degree of correlation between the scores for NK cells (r2 = 0.7, *p* = 1e−24) and naïve B cells (r2 = 0.8, *p* = 1e−56) from the two algorithms (see Supplementary Fig. 7).

**Table 3:** Differential composition and cell type enrichment by using two different deconvolution approaches.

(a) CIBERSORT differential cell composition				(b) xCell differential cell composition			
Cell types	logFC enrich	*P* Value	FDR	Cell types	logFC enrich	*P* Value	FDR
NK.cells.resting	−0.0215673	3.66e−04	8.05e−03	NK cells	−0.0030001	7.45e−03	6.81e−02
Neutrophils	0.0340265	1.11e−02	1.22e−01	Tgd cells	−0.0008546	9.08e−03	6.81e−02
B.cells.naïve	0.0084004	4.30e−02	2.68e−01	naïve B cells	0.0038983	3.69e−02	1.39e−01
T.cells.CD8	−0.0134798	5.95e−02	2.68e−01	CD4+ Tem	−0.0029231	3.70e−02	1.39e−01
Macrophages.M0	0.0006095	6.10e−02	2.68e−01	B cells	0.0064601	5.44e−02	1.63e−01

(a) On the left, using CIBERSORT with LM 22 base matrix.

(b) On the right, using xCell algorithm FDR correction with the Benjamini–Hochberg method.

### Effect of HLA alleles and KIR gene expression on NK cell proportion

Since a number of investigations has shown a potential relation between specific HLA allele groups and NK cell activity in the context of ASD^51–53^, we have applied a recently developed computational tool which allows to perform in silico HLA typing (reported in Supplementary Table 6), and use the inferred HLA genotype to create a personalized index to quantify HLA expression^32^. We have then tested the correlation between HLA allele expression and the enrichment score for NK cells obtained by XCell, focusing on the major HLA alleles for which expression was detected for more than 30% of the subjects. The results (supplementary Fig. 8a) suggest that the alteration in NK proportion has a poor correlation, and in most cases not significant, for every HLA allele tested. We explored in more details HLA-Cw7, an HLA allele previously associated with ASD^51^, but we could not detect significant differences in NK cell estimate within diagnostic categories based on specific allele groups (see Supplementary Fig. 8b) nor in allele frequency between ASD subjects and unaffected related siblings (see Supplementary Table 6).

Finally, since genes coding for activating Killer-cell immunoglobulin-like receptor (KIR) proteins influence NK cell activity, and their frequency has been found to be significantly increased in ASD^54,55^, we tested for the correlation between estimated NK cell proportion and the expression of KIR genes extracted from the original RNASeq dataset. The results (shown in Supplementary Figs. 9, 10) are consistent with the positive regulatory effect of KIR genes on NK cells, but do not suggest an increase frequency of specific KIR genes in ASD based on their expression. Therefore, putative differences in KIR gene frequency are unlikely to be at the basis of the observed altered NK cell proportion in ASD.

### DGE analysis with cell composition estimate as covariate

We hypothesized that part of the unexplained variance in the previous DGE model could be driven by altered immune cell composition in ASD and not uniquely by transcriptome dysregulation. Thus, including an estimation of cell composition in the model would enable to uncover dysregulated gene expression withstanding the systematic imbalance in blood cell proportion between the discordant sibling pairs. Based on the results obtained with xCell, we selected the most differential enriched cell types (*p*value < 0.05, i.e. NK cells, Tgd, naïve β cells and CD4+ Tem), and included their scores for each individual sample as additional covariates in the model. As a result, the number of significantly de-regulated genes was largely decreased compared with the standard model, and the inflation previously observed the drastically reduced (see QQ plot in Fig. 1b, lambda = 0.975). As shown in Table 2 (b), when accounting for cell composition, only three genes (PTPRN2 and HGMB and NMUR1) remained significantly different at FDR < 0.25 (see also Supplementary Fig. 11). We then compared our results with a recently published meta-analysis on brain gene expression in autism^56^. Notably, whilst for NMUR1 and PTPRN2 we could not identify a statistically significant signal, for our top finding, HMGB3, we found a significant downregulation in human brain tissues of autistic patients (FDR = 0.03), with consistent downregulation in both cortex and cerebellum (See Supplementary Fig. 12).

### WGCNA analysis

**Fig. 2:**
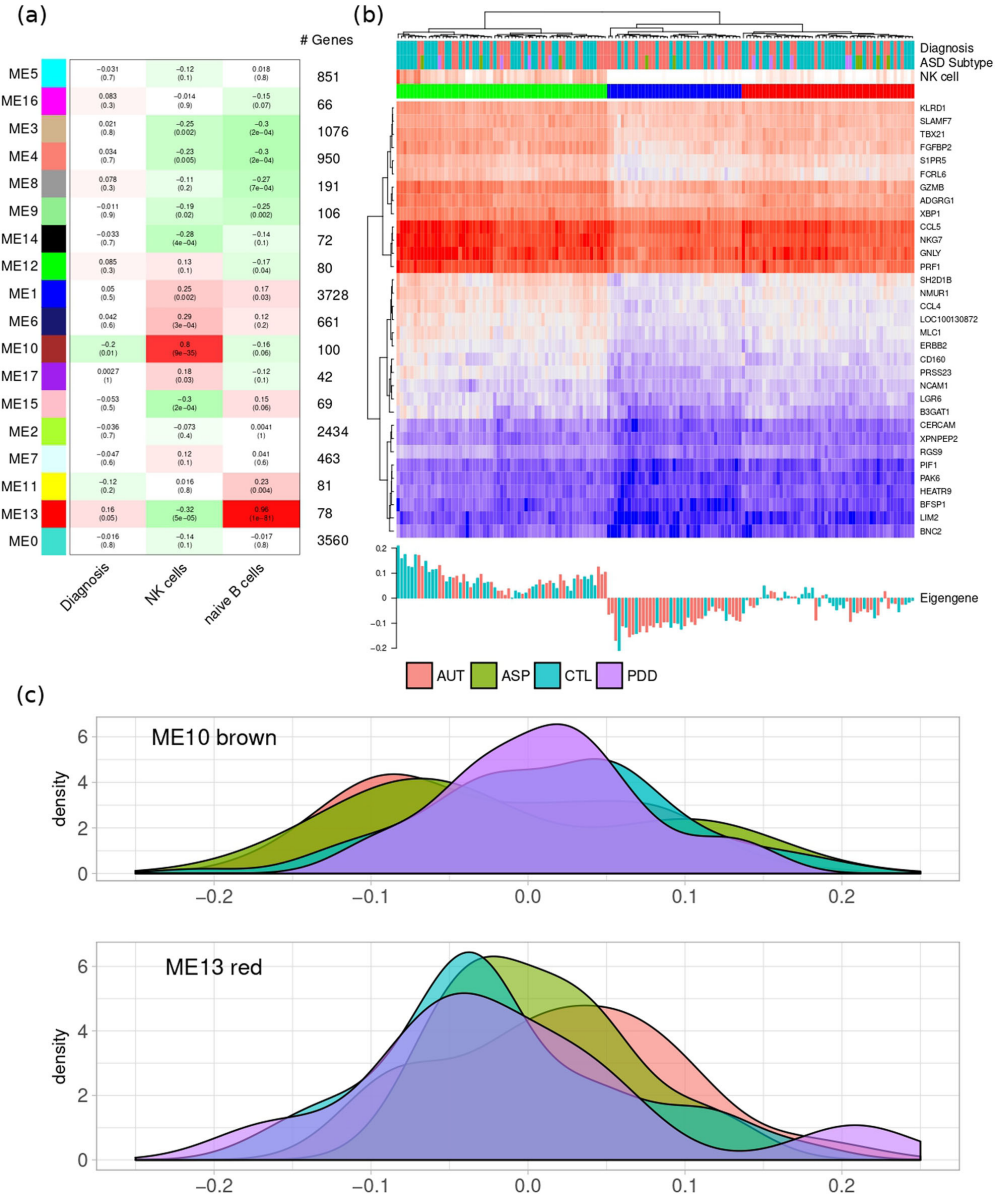
WGCNA Module analysis. **a** Module eigengene correlation with sample traits. **b** Expression heatmap for module 10—“brown”. At the bottom, the eigengene values for each sample. Hierarchical clustering on the top shows three groups for NK cell composition, with the right group enriched for ASD samples. **c** Distribution of the module eigengene for brown module 10 and red module 13. The top panel shows a shift of the distribution peak on the left for autism and Asperger, while PDD-NOS are closer to controls.

To corroborate the evidence of an effect of the imbalance in immune cell composition on the ASD gene expression signature, we conducted a further analysis based on WCGNA. Out of the network modules extracted by WGCNA, we identified two clusters of genes that are highly correlated with ASD diagnosis, and, at the same time, with the abundance of NK cell (ME10) or naïve Bcell (ME13) (see Fig. 2a). Whilst the expression heatmap constructed by using all expressed genes did not reveal any sample structure (not shown), the expression heatmap for ME10 obtained by unsupervised hierarchical clustering identifies three clusters, with the middle one (in blue) significantly enriched in ASD subjects (*p* < 0.003). The three clusters correspond to three groups with different NK cell proportion, as shown in Fig. 2b, top panel.

Interestingly, by looking at the distribution of the eigengene values for ME10 and ME13 across samples, differences between the three diagnostic subgroups can be observed (see Fig. 2c). In particular, a shift of the distribution peak for ME10 on the left was found for autism and Asperger, while PDD-NOS are closer to controls, suggesting that the decrease in NK cell proportion observed in ASD is not driven by PDD-NOS subjects. Similarly, the distribution of the eigengene values for ME13 is shifted towards the right for autism samples (but not for PDD-NOS and only marginally for Asperger), suggesting that the main difference in B cell composition is driven by autism subjects.

## Discussion

Although previous applications of RNASeq efforts have been described in an integrated analysis of blood transcriptome^57^ or in LCL^15^, our report, to the best of our knowledge, describes the largest differential gene expression profiling in peripheral blood of ASD subjects conducted by RNASeq. In this study, we exploited the availability of a well characterized family collection and a discordant sibling design to uncover peripheral transcriptional signatures for ASD. The high degree of kinship between siblings and the shared environment should minimize differences in transcriptome not strictly related to ASD. We have used a robust model accounting for numerous demographic and technical covariates, and we have found relatively subtle differences between ASD cases and unaffected siblings, with most variance in gene expression being driven by the family component. Nevertheless, we were able to identify a gene expression signature, with ten DEGs below FDR threshold of 0.05. Our results are consistent with most published gene expression investigations by microarrays in the blood or in blood cells of ASD subjects, where the number of DEG surviving to multi test correction is usually small, with some exceptions^21^. A recent meta-analysis^27^, reports a list of more than 1500 DEGs significant at FDR level, however a more accurate inspection shows a substantial inflation (with a calculated lambda of 1.87), likely due to unexplained variance, which is not corrected by SVA analysis (lambda = 2.32, ca 2300 DEGs). A cell-specific investigation in leukocytes of ASD subjects^25^ reports a list of ca 110 DEGs with FDR < 005, a relatively high number which might be somehow explained by the lack of covariates in the model. Indeed, when assessing differential expression in blood leukocytes in a sample of 118 subjects including part of the previous cohort, and adjusting for batch effects, sex, and RIN, no DEG resisted to multiple test correction for any pairwise group comparison^58^. A previous blood transcriptome analysis by microarrays on ASD discordant siblings reported the identification of a single gene with FDR < 0.05 after controlling for age, gender, and difference among families^22^, in line with our findings.

Enrichment analysis on our ASD signature suggests a dysregulation of immune-inflammatory pathways, consistently with previous evidence of immune imbalance in ASD^59–62^. In particular, the top-enriched pathways are NK cell mediated cytotoxicity, and Immunoregulatory interactions between a lymphoid and a non-lymphoid cell, which also involves receptors and cell adhesion molecules playing a key role in modifying the response of B, T, and NK cells to antigens and pathogenic organisms. Interestingly, when searching for disease gene sets associated with signature, we identified Cytomegalovirus infection as the top hit. CMV infections have been associated with ASD since long, and recent evidences suggest a high prevalence of congenital CMV infection in ASD cases as measured by PCR using dried blood spots on filter paper^63^. In the Italian population, the infection rate was found to be about 10-fold higher in ASD than in the general population^64^. Given the known involvement of NK cells in congenital CMV infection^65^, is it tempting to speculate a possible relationship between our findings and the increased prenatal exposure to viral infections, or other pathogens triggering similar mechanism, in ASD subjects.

The enrichment in immune-inflammatory pathways in blood gene expression signatures in ASD has been previously observed by other investigators^16,19,21,22,24^, and confirmed by two recent meta-analyses of previous studies^27,66^. Studies conducted on whole blood RNA extracted with our same procedure (PAXgene) have shown a deregulation of immunoinflammatory pathways, such as chemokine signaling and FC gamma R-mediated phagocytosis^21,22^. In particular, chemokine signaling was among the top downregulated pathways when comparing blood samples from 20 discordant sibling pairs of the Simons Simplex Collection^21^, consistent with our finding of a network of downregulated chemokines among top DEGs (See Supplementary Fig. 13). Of note, gene-expression classifiers of ASD identified based on microarray studies are enriched with genes related to immune response^19^ or immune/inflammatory functions^25^. The ASD signature identified by the most comprehensive meta-analysis of blood transcriptome investigations is also characterized by downregulation of innate immune and inflammatory signaling transcripts^27^, supporting the notion of overall decreased peripheral immune-inflammatory functions in ASD.

Deregulation of immune-inflammatory functions have been also reported in the brain of ASD subjects, in particular with the identification of an over-expressed module, named asdM16, enriched in astrocyte and activated microglial markers, and genes involved in immune and inflammatory responses^4^, which was related to “activated” M2 microglial and “immune response” genes by a subsequent RNASeq-based investigation in ASD cortical brain tissue^5^. More recently, in the largest transcriptome analysis of post-mortem brain tissue in ASD so far, upregulated modules in ASD were found to be enriched in genes associated with inflammatory pathways^6^. Different hypotheses have been postulated to explain why blood and brain signatures in ASD converge; however with opposite deregulation, on immunological functions, including the contribution of decoupling mechanisms, tissue-related differences in feedback response or post-mortem related mechanisms, warranting further investigations^27^.

To explore the immune cell repertoire in ASD subjects and estimate the effect of its dysregulation on the blood transcriptome, we exploited recent methods to infer cellular proportions from expression signatures of complex tissues. Using two complimentary methods, we were able to show a subtle, but statistically significant, decrease in NK cells paralleled by an increase in naïve B cells in ASD. The evidence from the cell deconvolution approach is corroborated by WGCNA analysis, which clearly identifies three clusters based on blood cell composition, possibly driven by different ASD subtypes.

Based on previous evidence of the effect of specific HLA allele groups and KIR gene variants on NK cell activity, we tested the hypothesis that differences in their frequency in ASD vs healthy controls could contribute to the alteration in NK cell proportion detected by cell deconvolution. We inferred HLA genotypes form RNASeq data and quantified expression of specific HLA alleles and KIR genes using recently developed pipelines. We could not correlate the alteration in NK cellular proportion between ASD and healthy siblings with any of the HLA alleles or KIR genes tested. Regarding HLA-Cw7, and other HLA allele groups having a role in stimulating NK cells, we could not find a significant difference in frequency between ASD subject and matched siblings in our collection, differently from Harville et al.^51^ who found an increased frequency in ASD vs unrelated subjects. Whilst we cannot rule out an increased overall increased activation of NK cell in ASD subjects, our data suggest a decrease in NK cell proportion over total blood cells according to signature deconvolution results irrespective from the HLA alleles or the KIR genes tested.

Our findings are consistent with the evidence of immune abnormalities in ASD, and the hypothesis of specific cellular immune-phenotypes related to different ASD subtypes^62,67^. Previous studies on NK cells in ASD, in particular on their proportion in blood and their activity, have produced mixed results, possibly due to the different CD markers used to determine cell counts. Two studies identified an increase in absolute number of NK cells in children with autism^17,68^, whilst one report provides evidence of a decrease in CD57+ NK cells^69^. On the other hand, when investigating NK cell cytotoxic activity ex-vivo, there seems to be a convergence on findings on a reduction of NK cell activity^17,68,70^, in agreement with early findings^71^. Of note, a recent work conducted in a group of adults with high functioning (hf) ASD reported no difference in the frequency of NK cells with respect to healthy controls; however, flow cytometric analysis revealed a hf-ASD signature based on NK cell-specific phenotypic markers, which was also associated with core ASD clinical dimensions^65^. Our work brings further evidence for a dysfunction in NK cell-related mechanisms in children with ASD. As suggested previously^17^, abnormalities in NK cells may play a role in ASD etiology by predisposing to adverse neuroimmune interactions and/or autoimmunity mechanisms during critical development periods. Likewise, we observed an increase in naïve B cells in ASD children compared with their unaffected siblings. Increase in mature or memory B cells in ASD children was previously reported^72,73^ even though most literature on B-cell number and function in ASD does not support significant abnormalities^62^.

Whilst numerous peripheral blood gene expression investigations have highlighted dysregulation in immunoinflammatory pathways in ASD^68^, little work has been conducted so far to investigate the consequence of immune cell imbalance in ASD signatures. In a recent transcriptome analysis of leukocytes in ASD, differences in proportion estimates of different leukocyte cell types were tested by using a deconvolution approach step^58^. No differences across diagnostic groups were identified; however, the authors used a different deconvolution algorithm and RNA extracted from whole blood after a cellular fractionation step^58^. A deconvolution analysis was also reported in a recent meta-analysis of blood ASD transcriptome investigations^27^, which suggest increased expression of genes specific to NK cells and T-helper cells, consistent with earlier finding by Gregg et al.^16^ showing gene expression differences in genes predominately associated with NK and CD8+ cells. The reported increase of NK cell-specific genes^27^ is not consistent with our finding of reduced NK cells component. We are unable to provide further insights on the above discrepancy, since details on the results of the deconvolution analysis are not reported for the meta-analysis^27^. Given that our findings have been supported by two independent approaches, the disagreement with the previous report is unlikely to be due to methodological issues, but rather to differences in the demographic characteristics of our cohort or in the sibling pair design.

Finally, to identify genes that are differentially expressed irrespective from ASD-related differences in the proportion of circulating blood cells, we included the cell composition estimates from deconvolution analysis as covariates in our generalized linear model. Three genes survived to the DGE analysis when conditioning to cell composition scores, namely HMGB3, PTPRN2, and NMUR1.

HMGB3 was significantly downregulated in our study as well in human brain tissues of autistic patients. HMGB3 is a member of the high mobility group superfamily, and it has never been reported to be related to ASD, although there are several evidences for a role of a member of the same family, HMGB1, in ASD-associated inflammation^74^.

We found a significant up-regulation of PTPRN2, coding for a Receptor-Type Protein Tyrosine Phosphatase N2, which is required for normal accumulation of secretory vesicles in hippocampus pancreatic islets and in the hippocampus^75^. PTPRN2 is also part of a set of protein complexes of which are tightly co-regulated during neuronal development^76^. A role for PTPRN2 in regulating brain development and function has been suggested, based on genetic disruptions linked to attention deficits, addiction and mood disorders, Down Syndrome and HOXA1 spectrum disorder^75^ suggesting a pivotal role in regulating brain development and function. More recently, copy-number variations in PTPRN2 have been identified in children with developmental coordination disorder^77^. Of note, PTPRN2 has been shown to be a target of autoantibodies in Type I diabetics and a regulator of insulin secretion^78^. Since intrauterine hyperglycemia and neonatal hypoglycemia have been shown to be a risk factor for ASD^79^, further investigations in cohorts with information on neonatal glycemia, maternal lifestyle or history of type I diabetes would be needed to test the hypothesis of a correlation between altered PTPRN2 expression in ASD and abnormalities in glucose or in prediabetes markers.

NMUR1 is the peripheral receptor for neuromedin, a neuropeptide with pleiotropic roles with multiple functions. In particular, a key role for neuromedin in regulating food intake, circadian rhythms and inflammatory response has been described^80^. Of note, NMUR1 has been found to be downregulated in children from mothers exposed to psychosocial stress during pregnancy, with concomitant methylation of an enhancer region in the NMR1 gene dependent on the maternal stress score and altered immune response at birth^81^. Since neuromedin U activation of NMUR1 elicits production of cytokines by T-cells^82^, further investigations can be envisaged to address a potential role for neuromedin U in mediating the interplay between neuroendocrine system and immune response in ASD.

Our study has a number of limitations and our results must be interpreted with caution. Although the discordant sibling design may help minimizing environmental or family-related confounders, our sample is rather heterogeneous, both in terms of ASD diagnosis and demographic factors, given our choice to include all available discordant pairs to maximize statistical power. Our statistical model, based on an empirical Bayes procedure, allowed us to estimate the beta coefficients for each technical and demographic covariate separately, including gender, ethnicity and diagnosis, resulting with a limited portion of unexplained variance, which can be fully ascribed to cell proportion according to QQ plot analysis. However, we cannot completely rule out subtle age, gender or ethnicity specific effects. This issue might be addressed by introducing gender-diagnosis, ethnicity-diagnosis or age-diagnosis interaction terms, which we opted out given the high complexity reached by of our generalized linear model. As far as gender is concerned, we have conducted a separate analysis for male sibling pairs, which corroborates our findings on the full cohort (the limited number of sibling pairs has hindered us to conduct a similar analysis on female subjects). Among demographic factors, a key element limiting the generalizability of our findings is the broad age range of our subjects, compared to some other investigations which have focused more specifically on toddlers or children within a narrow age range^19,24,25^. An Additional limitation is the lack of cell count data, which would have allowed an ultimate validation of our convergent findings of altered blood cell proportion in ASD, and placing full reliance on RNA Sequencing for the quantification of gene expression and for HLA genotyping. Finally, and more importantly, our findings should be seen as the result of an original investigation on a family cohort, which would need to be replicated in independent collections.

Despite the above limitations, taken together, our results suggest that subtle changes occur in the ASD blood transcriptome, which can be ascribed mainly to shifts in cellular composition, seemingly the major component driving gene expression changes when working with whole blood RNA. We identified three genes surviving cell deconvolution analysis which could be seen as novel suggestive biomarkers to be investigated in independent cohorts. Cell deconvolution analysis supports the hypothesis of a role of NK cells in the pathophysiology of autism, possibly related to increased prenatal exposure to infections in ASD subjects. While exploratory in nature, our findings are consistent with a growing body of evidence supporting immune-pathologies in ASD. The possibility of identifying subgroups with predominant immune system dysregulation, or to associate cellular immuno-phenotypes to different symptom dimensions might be a promising path forward in the development of non-invasive ASD biomarkers.

## Supplementary information

Supplementary Figures

Supplementary Tables
