# Supplementary material for: Transcriptome signatures from discordant sibling pairs reveal changes in peripheral blood immune cell composition in Autism Spectrum Disorder
Source: Transl Psychiatry. 2020 Apr 14;10:106. doi: 10.1038/s41398-020-0778-x (PMC7156413; doi:10.1038/s41398-020-0778-x)
Supplement: Supplementary file 1 — Supplementary Figures [file 41398_2020_778_MOESM1_ESM.pdf]

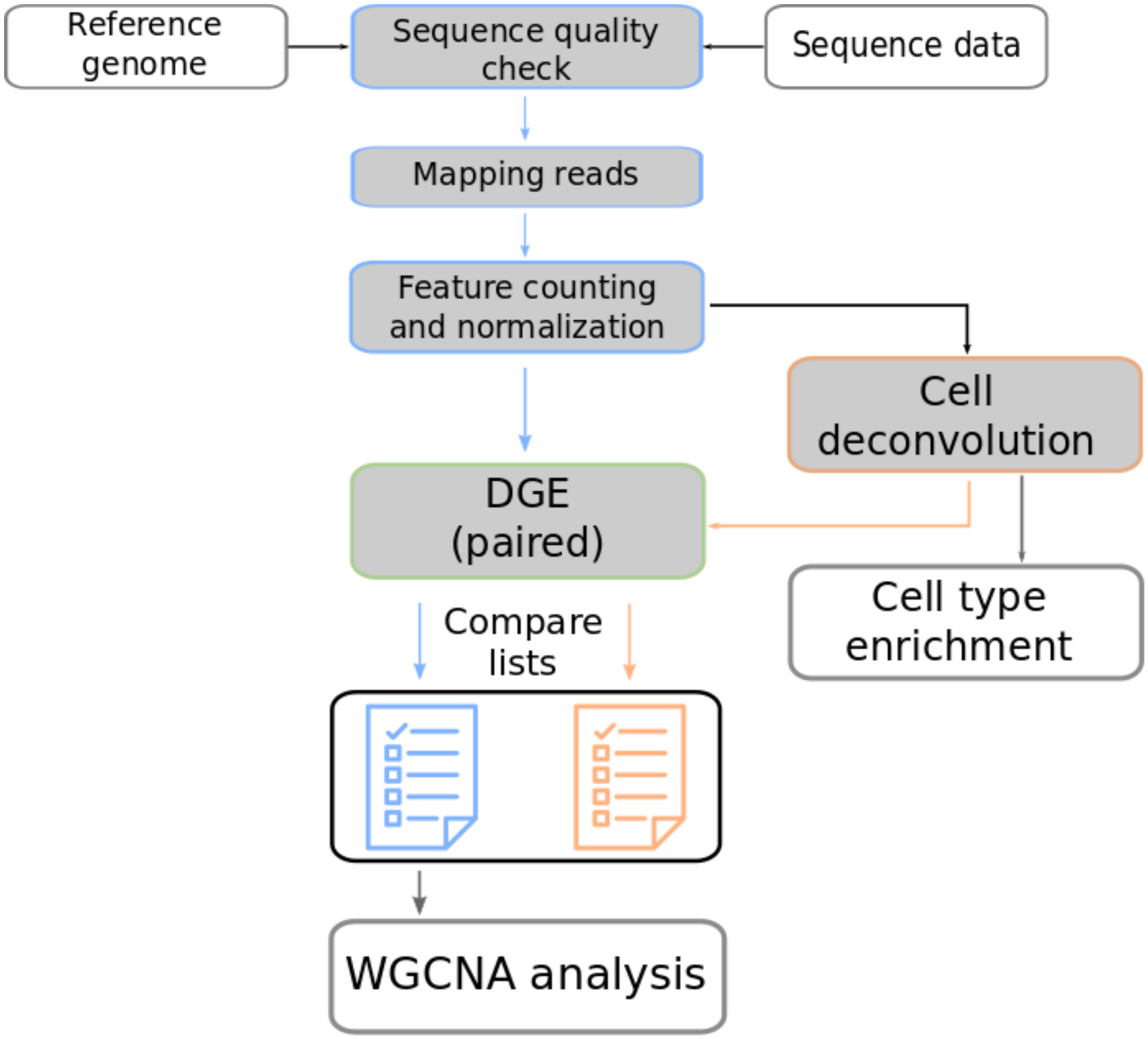

Figure S1: Data analysis pipeline for Differential Gene Expression analysis applied on the ITAN RNASeq dataset.

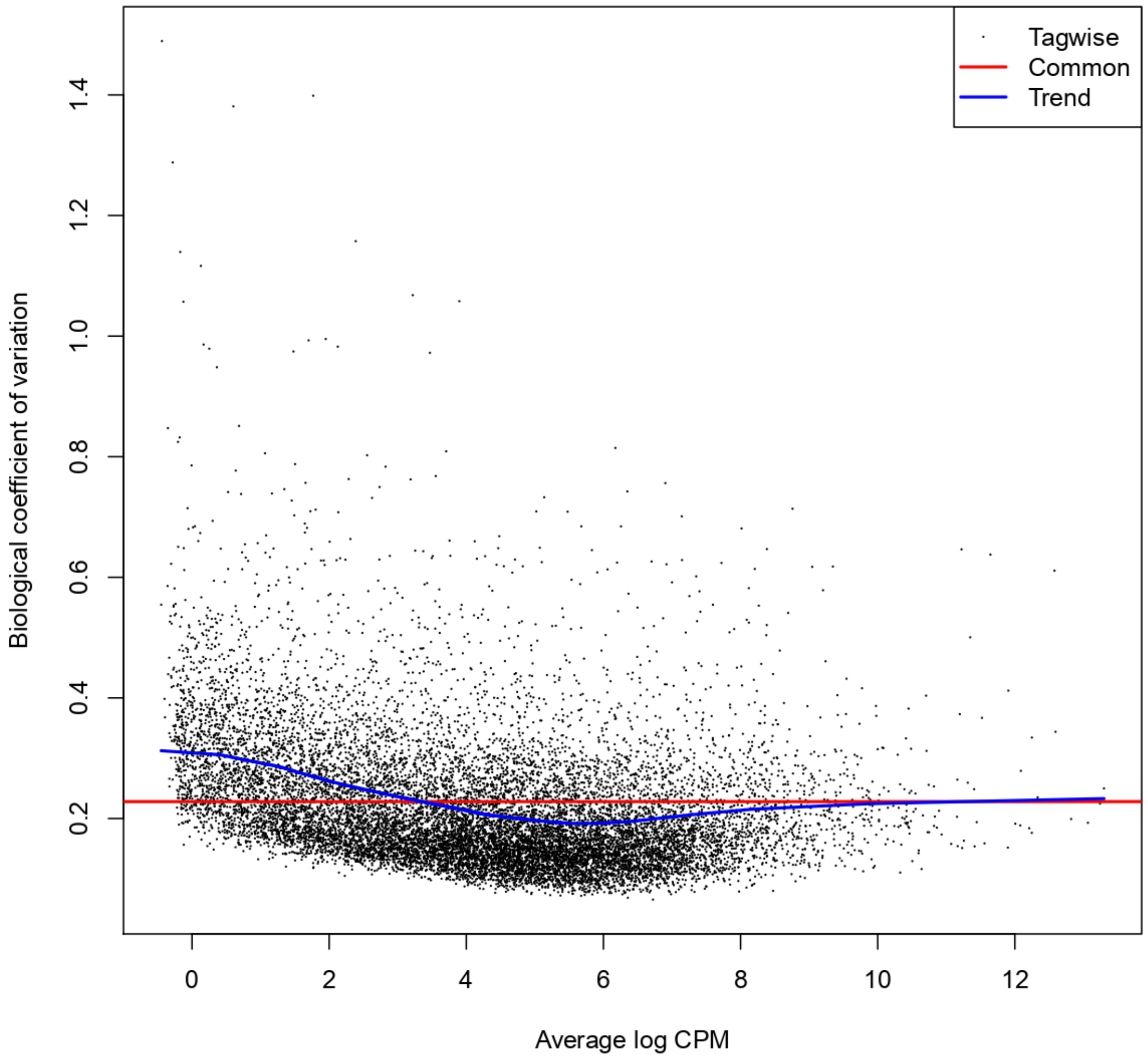

Figure S2: Estimation of the biological coefficient of variation used in the generalized linear model for DGE implemented in edgeR package.

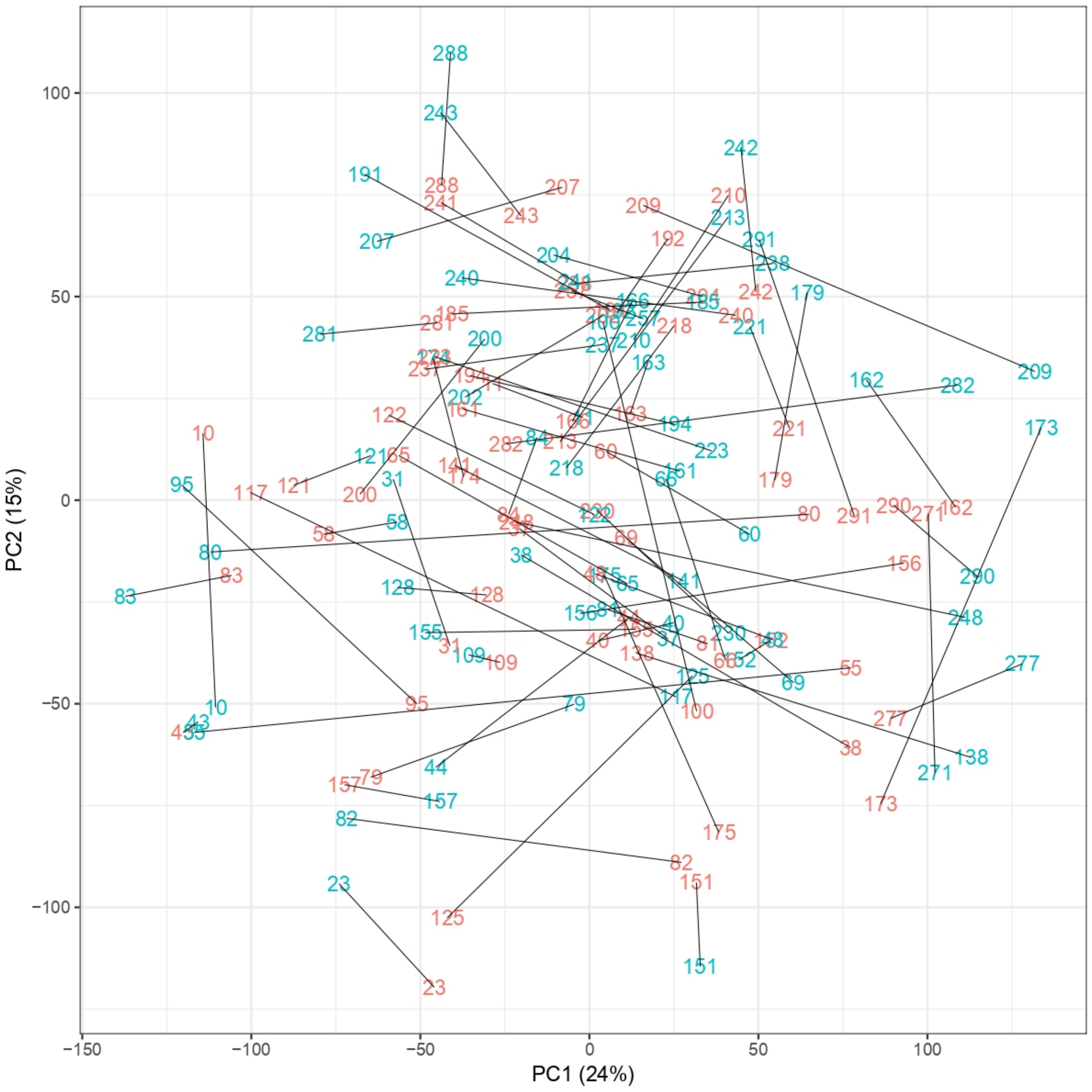

Figure S3: Principal Component Analysis on normalized gene counts, (Blue=ASD sibling, Red=Controls sibling, lines connecting siblings).

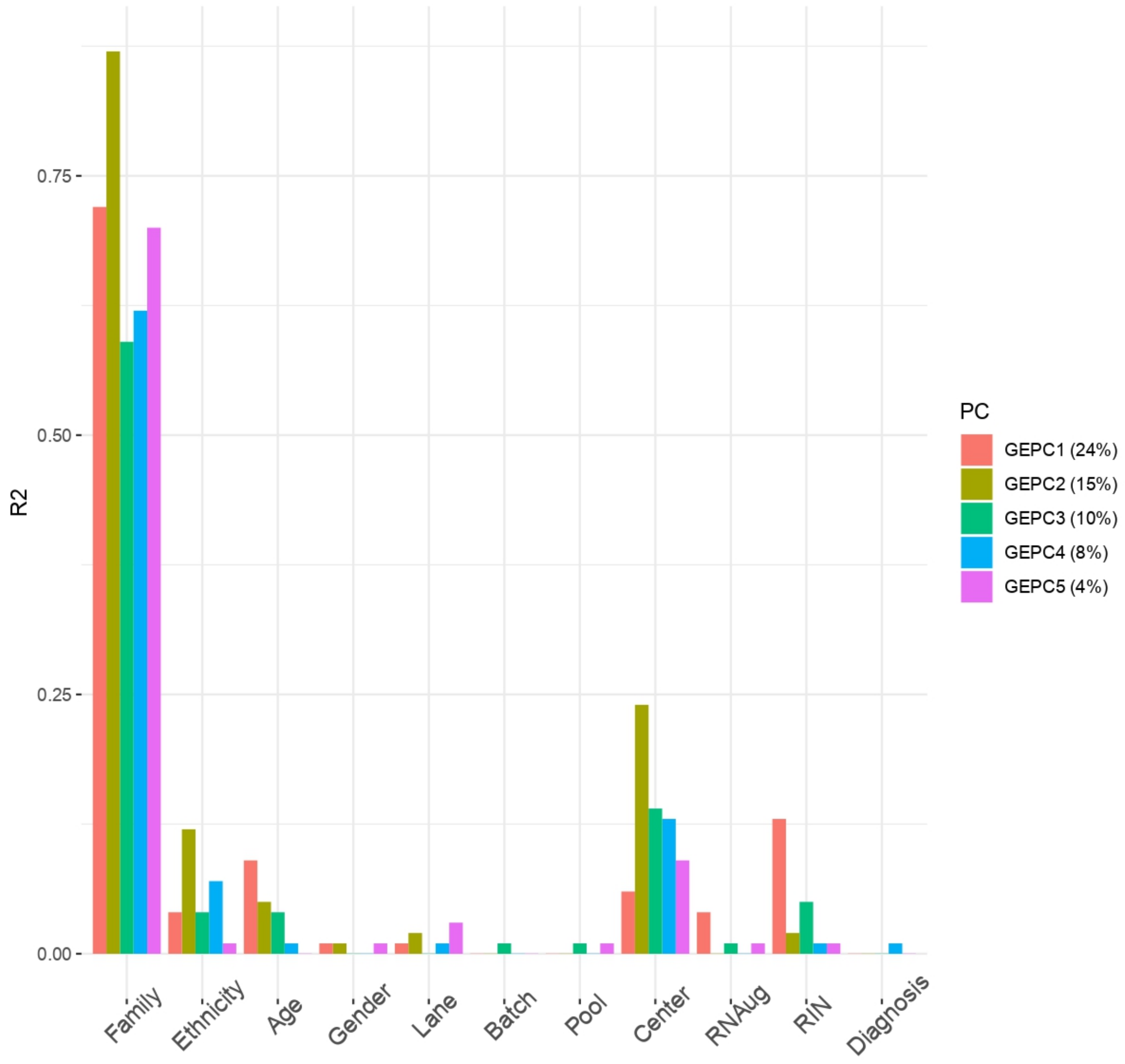

Figure S4: Variance explained by each available covariate for each of the first 5 principal components.

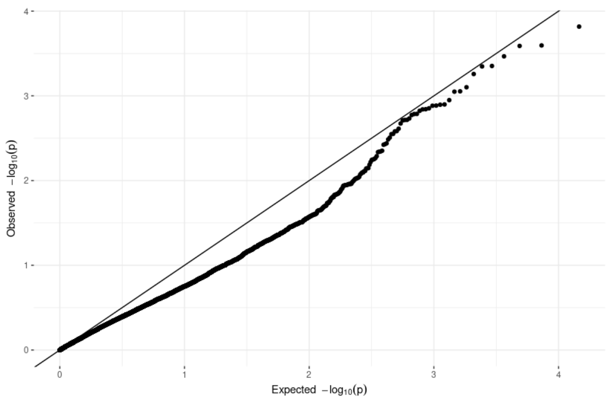

Figure S5a: DGE analysis on male samples only (with deconvolution covariate)

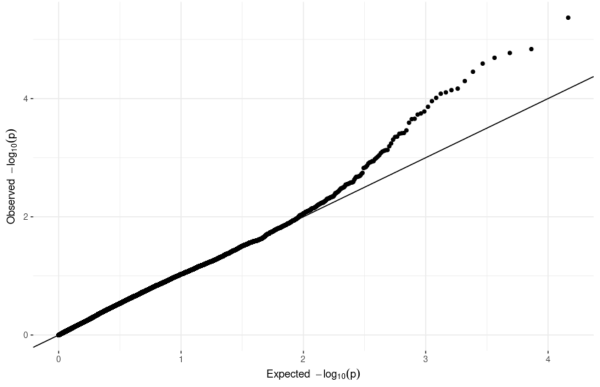

Figure S5b: DGE analysis on male samples only (no cell deconvolution covariate)

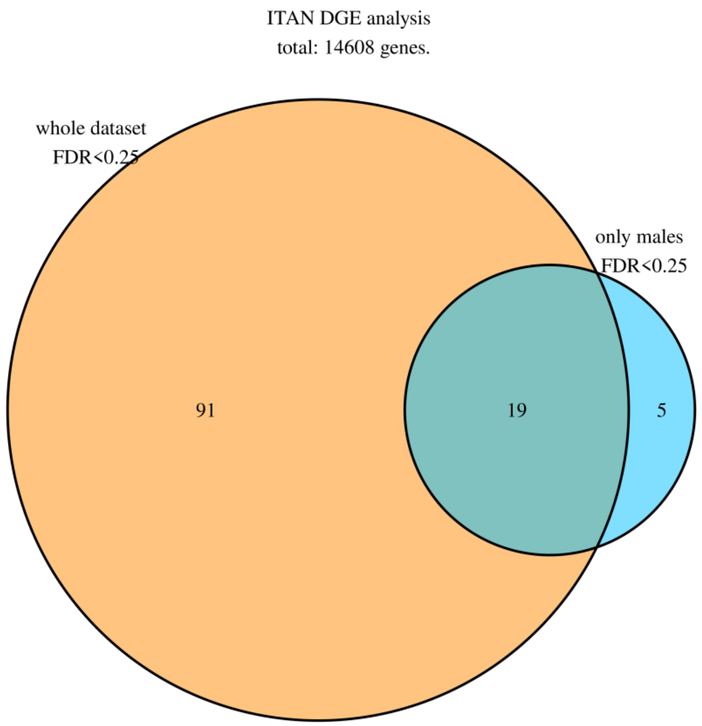

Figure S6: Venn diagram of the intersection between male DGE and whole dataset

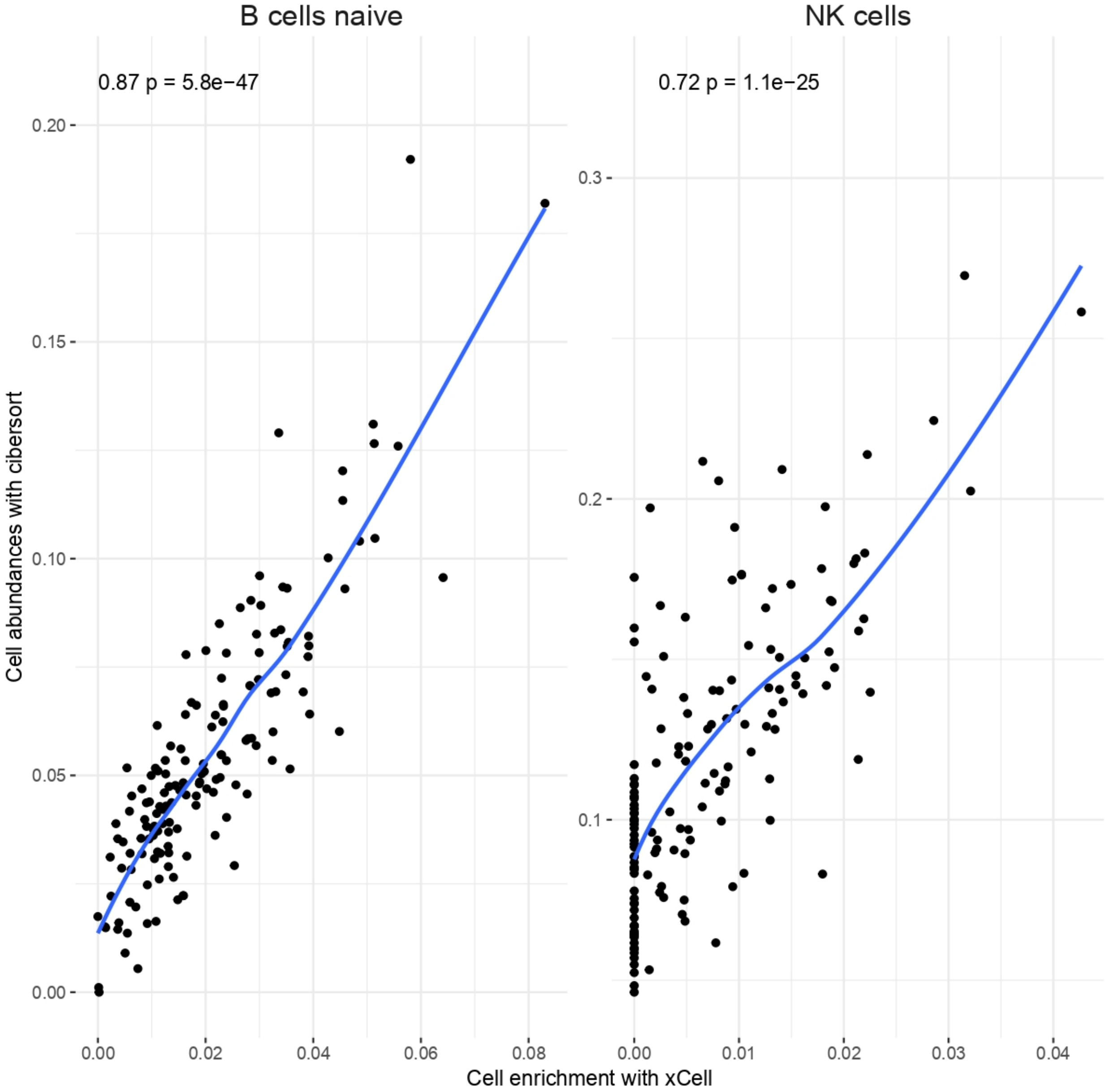

Figure S7: Correlation between cell estimates derived from different methods for cell deconvolution.

(a)

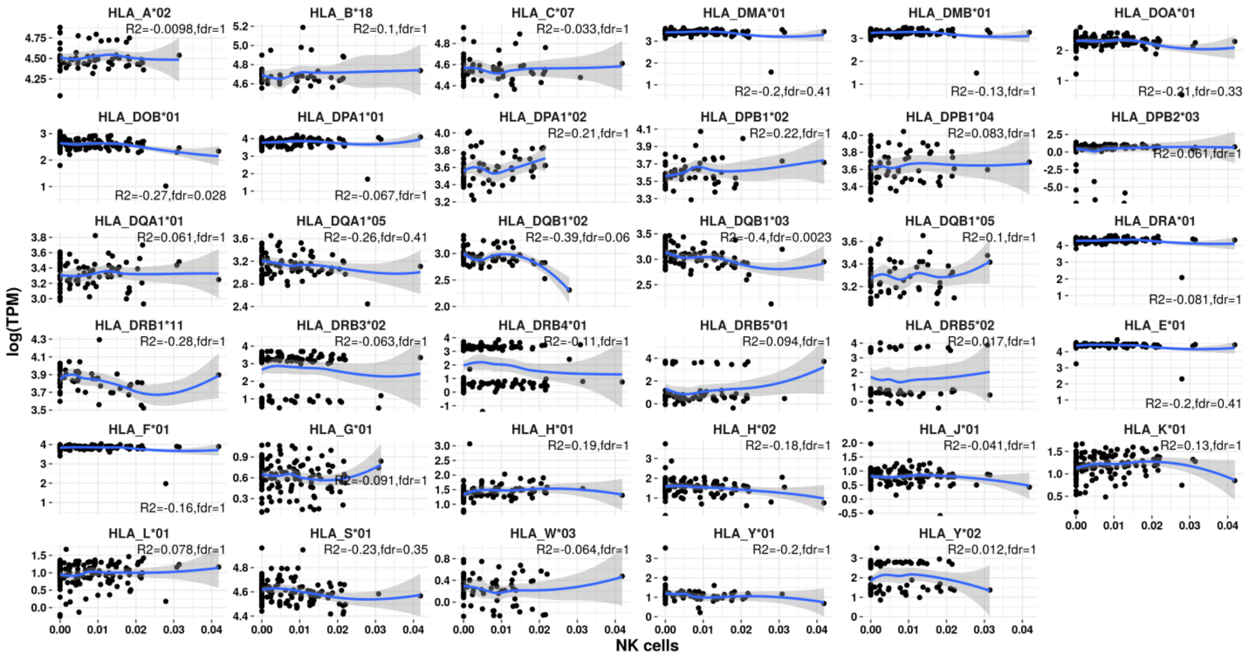

(b)

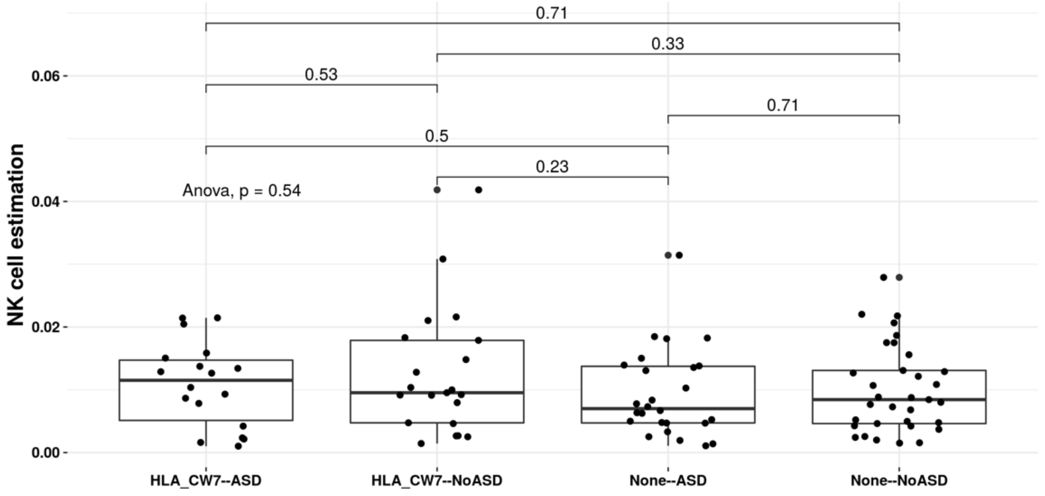

Figure S8: (a) Expression of HLA-allele transcripts (Transcripts Per Million, TPM) and correlation with xCell estimated NK cell enrichment. Only transcripts present in at least 30% of the samples are reported in the plots. (b) NK cell estimates within diagnostic categories based on presence/absence of HLA-Cw7. None of the comparisons have a significant p.value ($p<0.05$). Samples with a NK cell estimation score lower than 1e-6 have been removed from the plot.

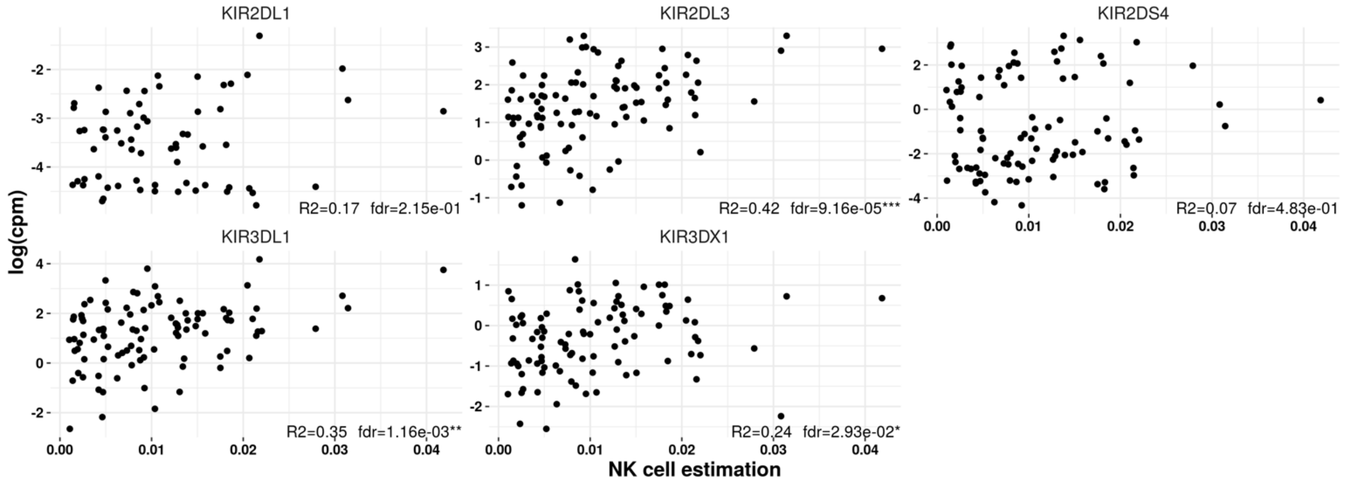

Figure S9: Correlation of expression (in log Count Per Million, CPM) for KIR genes from RNASeq and xCell enrichment score for NK cell abundance. Only transcripts with CPM > 1 in at least 30% of the samples were included in the plot. The significant correlation found for KIR2DL3, KIR3DL1 and KIR3DX1 supports the notion of a positive regulatory effect of KIR genes on NK cells

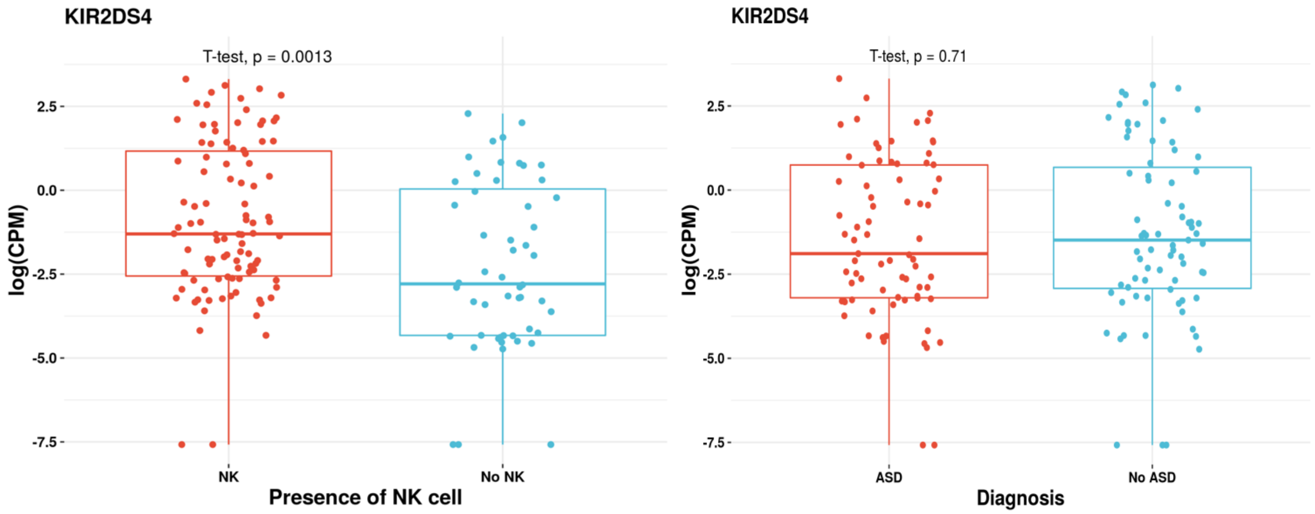

Figure S10: KIR2DS4 expression in log Count Per Million reads (CPM) estimated by RNASeq grouped by presence/absence of NK cell (based on a threshold of NK cell enrichment score > 10E-06), left panel. The difference observed support the NK cell activating role of KIR2DS4. Nevertheless, KIR2DS4 expression is not significantly different between ASD cases and unaffected controls (right panel).

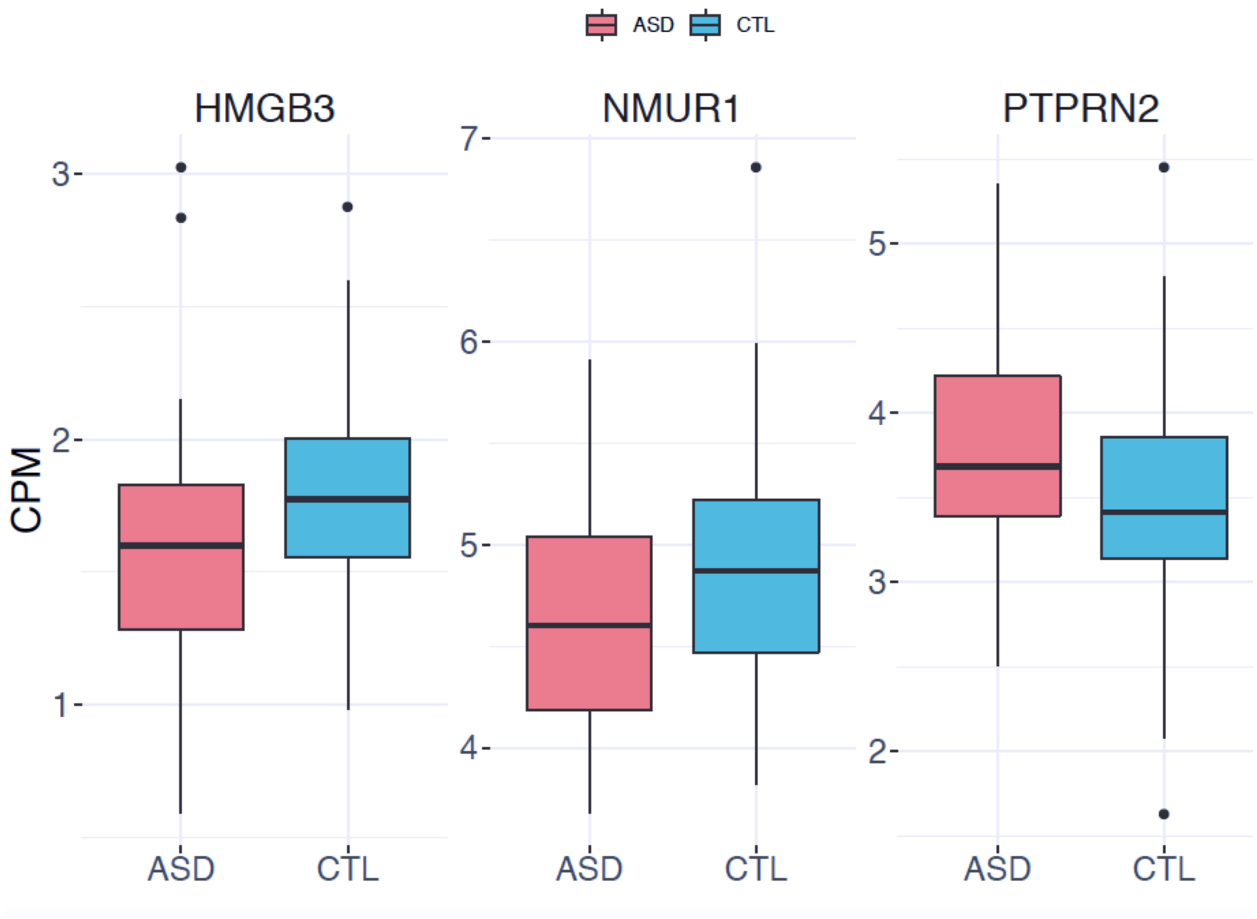

Figure S11: Gene expression signature differences between discordant siblings for statistically significant genes (at FDR<0.25) after accounting for cell composition.

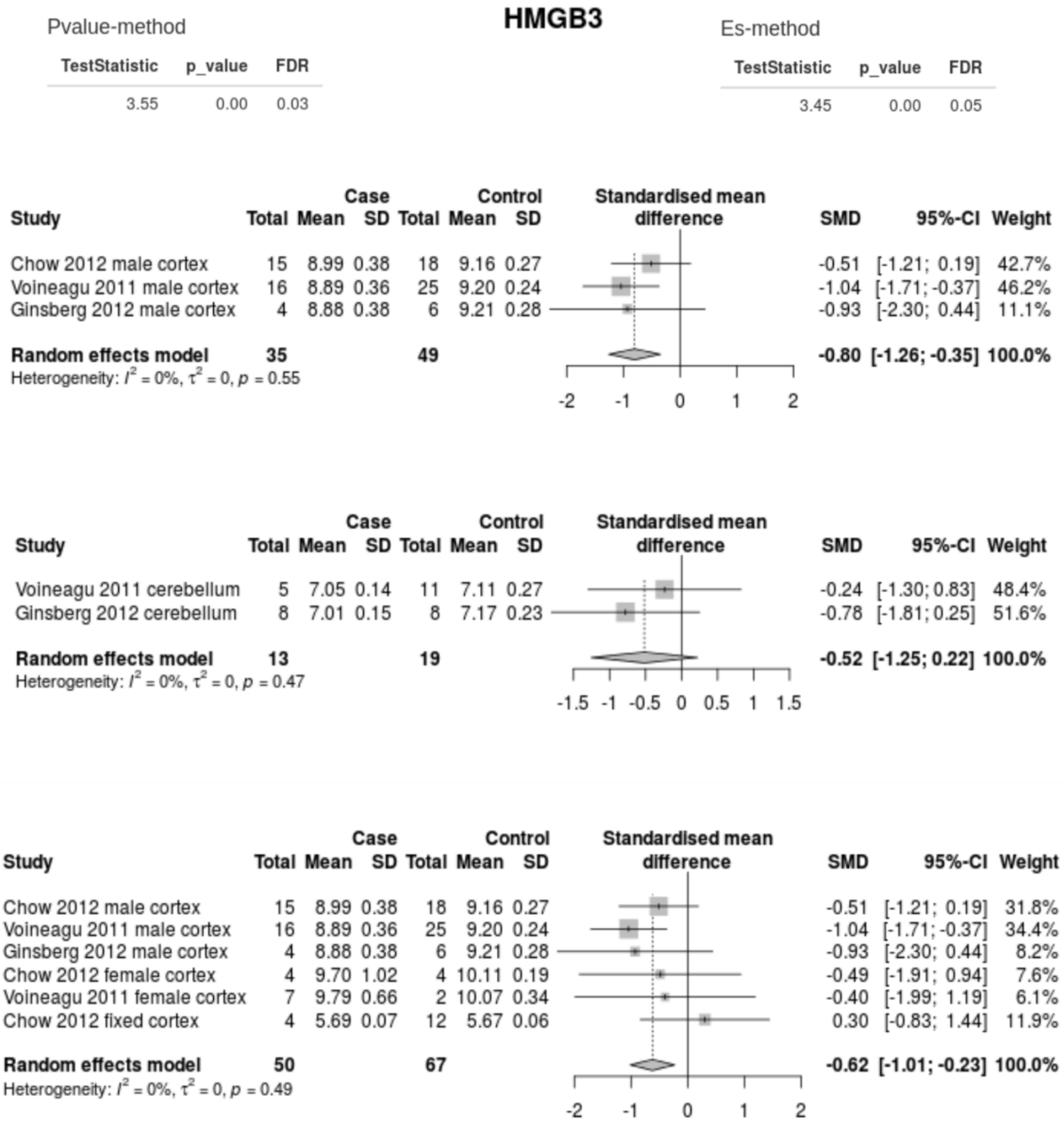

Figure S12: Expression profiles of HMGB3 in brain tissue from human ASD studies extracted from dbMEGA (meta-analysis of ASD databases, https://dbmdega.shinyapps.io/dbMDEGA/, Zhang et al., 2017)

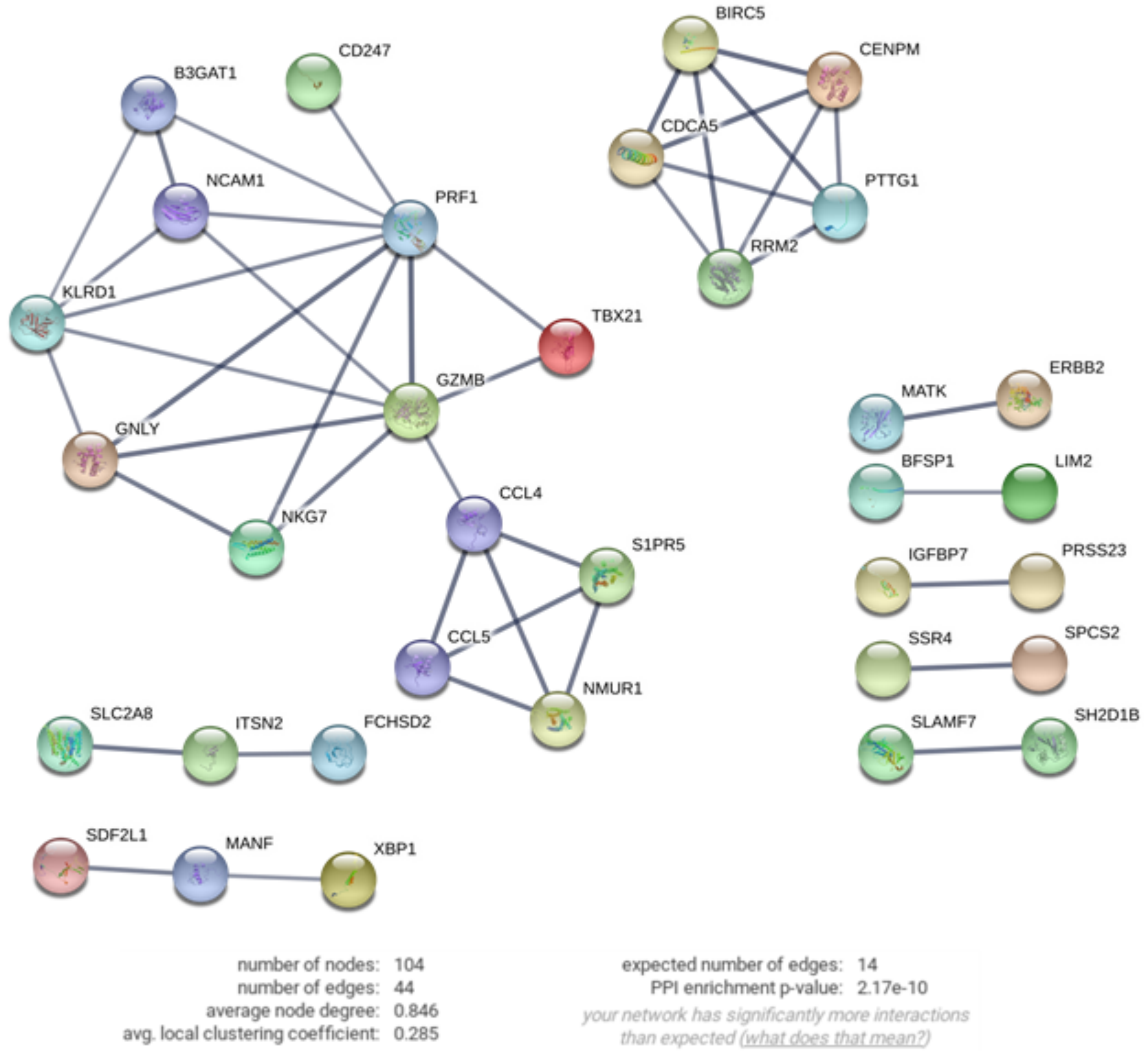

Figure S13: Enrichment on STRING protein-protein interaction database of the list of differentially expressed genes with FDR <0.25. All available interaction sources have been used to infer confidence of interaction between the submitted genes. Only links with a confidence of 0.7 or more are shown in the graph. Nodes without connection have been removed from the plot.
